# Recurrence of dysphagia post-myotomy: etiologies and management

**DOI:** 10.1590/0100-6991e-20202973

**Published:** 2021-03-31

**Authors:** MARINA FELICIANO ORLANDINI, WANDERLEY MARQUES BERNARDO, FRANCISCO TUSTUMI

**Affiliations:** 1 - Centro Universitário Lusíada, Departamento de Medicina Baseada em Evidências - Santos - SP - Brasil; 2 - Universidade de São Paulo, Departamento de Medicina Baseada em Evidências - São Paulo - SP - Brasil; 3 - Hospital Israelita Albert Einstein, Departamento de Cirurgia - São Paulo - SP - Brasil

## To the Editors

We read with great esteem the original article written by Costa et al^1^. In their study, the authors describe their experience with patients with megaesophagus who, after previous cardiomyotomy, had recurrence of achalasia symptoms / signs, especially dysphagia. They reported that the average time between the two surgical interventions, corresponding to the duration of the dysphagia, was nine years, and that these patients went through an average of 7.36 endoscopic dilations during this period, in unsuccessful attempts to improve the symptoms / signs.

Heller myotomy is known to be the most used megaesophagus treatment for most patients. After myotomy, the persistence or recurrence of symptoms / signs is 10-20%, the most common of them being dysphagia, regurgitation, aspiration, and heartburn^2^. However, for a more appropriate management of cases of recurrence of symptoms / signs after myotomy, it is essential to investigate the hypotheses. To optimize the diagnostic and therapeutic management, we can classify the symptoms / signs in three categories: “persistence”, “early recurrence”, and “late recurrence”.

In the case of persistent symptoms / signs, there has usually been a technical failure of the surgical intervention. In such conditions, the search for technical flaws can be done initially by evaluating the surgery video, if available. A common technical failure is incomplete myotomy, when done in a limited way, without involving the entire musculature of the lower esophageal sphincter. Moreover, too tight crurorraphy or fundoplication, leading to constriction of the esophagogastric junction, may even worsen dysphagia^3^. Such complications can be investigated by performing a video-esophagogram, which will demonstrate the contrast progression failure in the region of the fundoplication or hiatoplasty. Once an incomplete myotomy is diagnosed, the patient can undergo laparoscopic remiotomy. When the patient has undergone myotomy with associated fundoplication, perioral endoscopic myotomy (POEM) is an interesting alternative, since it avoids the lysis of adhesions necessary for a new Heller myotomy, and the patient will not have an exacerbated risk of gastroesophageal reflux, the most common complication of POEM^3^ ^,^ ^4^. Endoscopic dilation is an interesting alternative and can even serve as a therapeutic trial. If the patient does not show any improvement with dilation in these situations, it is possible that fundoplication or hiatoplasty are causing constriction in the esophagogastric transition, a failure that cannot be resolved with endoscopic therapy, and reoperation is indicated. If the dysphagia is mild, one should consider the possibility of transient dysphagia, which is common when there is mobilization of the distal esophagus^5^.

In early recurrence, a possible cause of reappearance of symptoms / signs is valve migration, ie the transdiaphragmatic herniation of the fundoplication, be it partial or complete. In this case, the most common symptoms / signs in the recent postoperative period are chest pain, dysphagia, and vomiting. Often such symptoms / signs are acute and there is strong association with increased intraabdominal pressure, which increases the risk of valve migration^6^. The investigation of the condition should be done through the esophagogram, visualizing the gastric fundus above the diaphragmatic crura, with narrowing of contrast passage^7^. Once migration of the valve is diagnosed, a repair surgical procedure must be carried out as early as possible, to prevent tissue necrosis.

Late dysphagia may represent an obstructive mechanical factor or the progression of the megaesophagus. Under these conditions, endoscopy is performed to identify the presence of mechanical obstructions, such as impacted, poorly digested food. Furthermore, endoscopy with biopsy is useful to investigate esophageal neoplasia, since achalasia is a known risk factor for its development, mainly squamous cell carcinoma^8^. Neoplasia can trigger dysphagia recurrence years or even decades after myotomy^8^. Gastroesophageal reflux inducing peptic stenosis is an uncommon complication, but it can occur, especially in myotomy operations without associated fundoplication^2^. If the endoscopy does not show mechanical obstruction, an esophagogram should be performed to investigate whether there is progression of megaesophagus dilation. After years, the esophagus can increase its caliber and lose its axis, assuming the sigmoid or dolichomegaesophagus aspect. In these circumstances, one should consider esophagectomy^9^. A new myotomy associated with rectification of the distal esophagus can be contemplated in patients with mild symptoms and a significant number of comorbidities that render esophagectomy unsafe, thus avoiding the risks associated with it, such as anastomotic leak, laryngeal nerve injury, bleeding, and chylothorax^10^.

Recurrence of achalasia symptoms / signs after Heller myotomy is not uncommon. The time of symptoms / signs reappearance, associated symptoms, and data provided by available tests assist in the investigation and determination of the etiology and guide the appropriate management of patients with recurrence of symptoms after Heller myotomy.
